# Distinct morphology of cardiac‐ and brown adipose tissue‐projecting neurons in the stellate ganglia of mice

**DOI:** 10.14814/phy2.15334

**Published:** 2022-05-27

**Authors:** Madeleine S. Barrett, Deborah M. Hegarty, Beth A. Habecker, Sue A. Aicher

**Affiliations:** ^1^ 6684 Department of Chemical Physiology & Biochemistry Oregon Health & Science University Portland Oregon USA

**Keywords:** cholera toxin B, dendritic arbors, immunocytochemistry, sympathetic, tract tracing

## Abstract

Sympathetic neurons that innervate the heart are located primarily in the stellate ganglia (SG), which also contains neurons that project to brown adipose tissue (BAT). These studies were designed to examine the morphology of these two populations (cardiac‐ and BAT‐projecting) and their target connectivity. We examined SG neurons in C57BL/6J mice following injections of the retrograde tracer cholera toxin B (CTb) conjugated to Alexa Fluor 488 and Alexa Fluor 555, into cardiac tissue and intrascapular BAT. BAT‐projecting SG neurons were widely dispersed in SG, while cardiac‐projecting SG neurons were localized primarily near the inferior cardiac nerve base. SG neurons were not dual‐labeled, suggesting that sympathetic innervation is specific to the heart and BAT, supporting the idea of “labeled lines” of efferents. Morphologically, cardiac‐projecting SG somata had more volume and were less abundant than BAT‐projecting neurons using our tracer‐labeling paradigm. We found a positive correlation between the number of primary dendrites per neuron and soma volume in cardiac‐projecting SG neurons, though not in BAT‐projecting neurons. In both SG subpopulations, the number of cholinergic inputs marked with vesicular acetylcholine transporter (VAChT) puncta contacting the soma was positively correlated to soma volume, suggesting scaling of inputs across a range of neuronal sizes. In separate studies using dual tracing from left and right BAT, we found that BAT‐projecting SG neurons were located predominately ipsilateral to the injection, but a small subset of SG neurons project bilaterally to BAT. This tracing approach will allow the assessment of cell‐specific mechanisms of plasticity within subpopulations of SG neurons.

## Graphical abstract

We have examined the morphology and the circuitry of brown adipose tissue‐ and cardiac‐projecting neurons in the stellate ganglia (SG) using retrograde tracer injections of cholera toxin subunit B (CTb) conjugated to two different fluorophores. This dual tracing of two sympathetic subpopulations of the SG is a novel method to study multiple cell groups based on their targets. We have shown that BAT‐ and cardiac‐projecting SG neurons are separate, non‐overlapping neuronal populations with distinct features through advanced confocal imaging, immunocytochemistry, and image analysis.

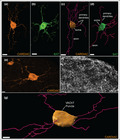

## 1 INTRODUCTION

The stellate ganglion (SG) contains sympathetic neurons that innervate a variety of tissues including the heart and intrascapular brown adipose tissue (BAT). Previous studies have begun to characterize the number, distribution, and innervation patterns of SG neurons in several species (Francois et al., 2019; Rajendran et al., 2019). However, these studies often use retrograde labeling techniques to study a single neuron subpopulation per animal. By studying multiple SG neuron subpopulations based on their target organs, direct structural and functional comparisons can be made between distinct SG neuron groups.

In this study, we use dual retrograde tract tracing to explore the distribution and innervation pattern of SG neurons to the heart and BAT. This approach reveals differences in cell distributions within the SG, differences in connectivity and laterality of efferent projections, as well as morphological differences in SG cells based on tissue target. To best visualize neuron morphology, our method used cholera toxin subunit B (CTb) fluorophore conjugates, which yield superior dendritic and somatic labeling compared to other tracers such as Fluoro‐Gold and fluorescent beads. Additionally, the multi‐fluorophore capability of CTb tracers makes it especially suitable for this study.

Preceding studies, primarily in larger species, have characterized the morphology of sympathetic post‐ganglionic neurons in other paravertebral ganglia (Andrews et al., 1996; Jobling & Gibbins, 1999; Purves & Lichtman, 1985; Ruit et al., 1990) besides the SG. These studies found morphological differences between sympathetic neuronal subpopulations, but without dual tract tracing. By studying neuron subpopulations that have been specifically labeled according to their targets, we are able to make inferences about the origins of neurotrophic signaling. Additionally, the SG is of particular interest as it has a large population of cardiac‐projecting neurons relative to other sympathetic ganglia. Our dual tracing method allows for examination of the heterogeneity between two SG subpopulations innervating different tissue targets (the heart and BAT).

## 2 MATERIALS AND METHODS

### 2.1 Experimental animals

C57Bl/6J mice acquired from The Jackson Laboratory (8 males and 2 females, 28–32 weeks, 24–39 g) were used for tracer injections. Mice were housed in 12 h light/dark cycles with continuous access to food and water.

### 2.2 Tract tracer injections

Mice were anesthetized with isoflurane (4% to induce, 2% maintenance in 100% oxygen), and core body temperature was maintained via feedback rectal sensor (37°C) connected to a heating pad (RightTemp Jr., Kent Scientific). Lactated ringers (0.5 ml) were given subcutaneously, hair was removed from the surgical area, eye lubrication was applied, and the incision site was sterilized with 70% isopropyl alcohol and 10% povidone‐iodine.

#### 2.2.1 General surgical procedures

Cholera Toxin Subunit B (CTb) tracers conjugated with Alexa Fluor (AF) 488 or AF 555 (0.1% in 0.01 M PBS, Invitrogen ThermoFisher Scientific C22841 and C22843) were injected into sites of interest using a Hamilton syringe (see details below). After tracer injection, the injection site was blotted with a cotton swab; 6–0 suture (Covidien) and tissue glue (VetBond) were used to close incisions; 5% lidocaine cream was applied topically. Analgesics were administered subcutaneously (meloxicam 5 mg/kg and buprenorphine SR 0.1 mg/kg). Once righting and twitch reflexes were recovered, mice were monitored in a heated, oxygen‐supplied recovery chamber until they were returned to their home cage. Mice were housed individually and monitored daily following surgery.

#### 2.2.2 Cardiac injections

Mice were intubated and mechanically ventilated for cardiac tracer injections, following the above general surgical procedures. A left thoracotomy was performed at the third or fourth intercostal space and 10 µl of tracer was injected through the pericardial sac, similar to a method described in a study by Rajendran et al., 2019. After wound closure (see above), mice were weaned off the ventilator slowly as reflexes returned.

#### 2.2.3 Brown adipose tissue (BAT) injections

Following the general surgical procedures described above, anesthesia was maintained via nose cone for BAT injections. BAT was exposed via a vertical incision between scapulae and left and right intrascapular BAT each received injections at two sites with 2 µl of CTb tracer, for a total of 4 µl per side. Closing and recovery followed the general surgical procedures outlined above.

### 2.3 BAT‐projecting SG neurons and time course analysis (Experiment 1)

**FIGURE 1:**
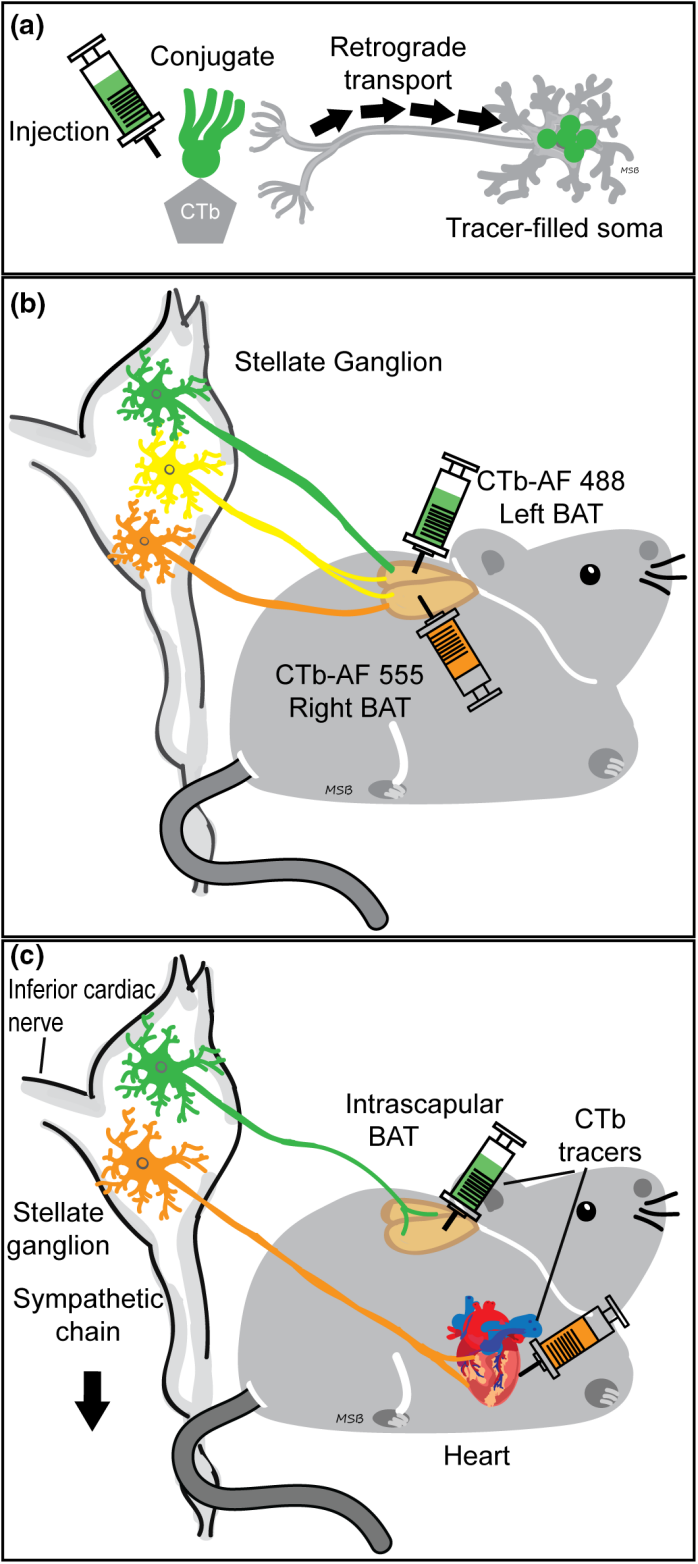
Experimental design: Dual tracing methods retrogradely labeled different sympathetic neuron stellate ganglia (SG) subpopulations based on the tissue injected. (a): Schematic showing cholera toxin subunit b (CTb, Invitrogen) retrograde tracer uptake from axon terminals near injection site to somata, where the fluorescent conjugate (green) was detected. (b): Left and right intrascapular brown adipose tissue (BAT) dual tracer injections retrogradely labeled SG BAT‐projecting neurons. Using different tracer conjugates on each side allowed us to study the circuitry of the BAT‐projecting SG population, including bilateral projections (yellow). We also examined tracer viability at multiple post‐injection times (7, 14, and 28 days). (c): Dual tracer injection techniques allowed for direct comparison and quantification of two neuronal subgroups within SG: Intrascapular BAT projecting (green) and cardiac projecting (orange).

In Experiment 1, mice were injected with two different CTb tracer conjugates (Alexa Fluor 488 and 555) in left and right BAT sites with tracers counterbalanced between left and right sides across animals (Figure 1a,b). Mice were anesthetized for 15–20 min to complete BAT tracer injections. Groups of mice were examined at different times (7, 14, and 28 days) following injections to assess tracer viability over time.

### 2.4 Dual‐labeling and tissue target collateral analyses (Experiment 2)

In Experiment 2, mice received a cardiac tracer injection followed by bilateral BAT tracer injections (Figure 1a,c). Mice were anesthetized for 90–105 min to complete both tracer injections. A different CTb tracer conjugate (AF‐488 or AF‐555) was used at each tissue site (BAT or cardiac), and tracers were counterbalanced between mice. Pre‐ and post‐operative procedures were as described above.

### 2.5 Stellate ganglia (SG) dissections

Stellate ganglia (SG) were harvested at 7, 14, and 28 days post‐injection for mice in Experiment 1, and at 7 days for Experiment 2. Mice were deeply anesthetized with isoflurane and then euthanized by decapitation. Left and right stellate ganglia (SG), located lateral to the longus colli muscles, across the second rib (Scherschel et al., 2020), preserving the inferior cardiac nerve (iCN) branch, were removed. SG were immediately fixed in 4% paraformaldehyde (PFA), 0.1 M phosphate buffer (PB) for 30 min, rinsed in 0.1 M PB and stored in cryoprotectant (30% sucrose/30% ethylene glycol in PB) at −20°C until processing.

### 2.6 Immunocytochemistry

The following process was based on previously published procedures in the lab (Aicher et al., 2013; Hegarty et al., 2018). Prior to immunocytochemical processing, fixed stellate ganglia (SG) were rinsed with 0.1 M PB followed by 0.1 M Tris‐buffered saline pH 7.6 (TS) and placed in 0.5% bovine serum albumin (BSA, Sigma‐Aldrich) in TS for 30 min. SG were then incubated for 48 h at 4°C in primary antibody solution of goat anti‐cholera toxin b (CTb) (1:25000, cat. 703, List Biological, RRID: AB_10013220) (Experiment 1), or goat anti‐vesicular acetylcholine transporter (VAChT) (1:1000, cat. ABN100, EMD Millipore, RRID: AB_2630394) (Experiment 2), in 0.25% Triton X‐100 (Sigma)/0.1% BSA in 0.1 M TS. SG were then rinsed and incubated for 2 h at room temperature in donkey anti‐goat Alexa Fluor (AF) 647 secondary antibody (1:800, cat. 705–605–147, Jackson ImmunoResearch, RRID: AB_2340437) in 0.1% BSA in TS. SG were rinsed, whole mounted, and cover‐slipped using Prolong Gold Antifade mountant (ThermoFisher). The goat anti‐CTb antibody has been used extensively to detect the beta subunit of cholera toxin (Hegarty et al., 2010, 2018) and preadsorption of this antibody with CTb abolished immunolabeling in rat and rabbit spinal cord sections (Llewellyn‐Smith et al., 1995). The VAchT antibody has been used extensively in recent rodent studies to identify cholinergic neurons using immunocytochemistry (Avila et al., 2020; Webber et al.,) and VAchT protein levels using Western blotting (Li et al., 2021; Yokoi et al., 2021).

### 2.7 Confocal imaging and analysis

#### 2.7.1 Cell count analysis

To examine cell counts and distribution, confocal images were taken of whole stellate ganglia (SG). Images were acquired using a Zeiss LSM 900 confocal microscope with a Plan‐Apochromat 20x/0.8 M27 objective in the Advanced Light Microscopy Core (ALMC) at OHSU. Six 1024 × 1024 pixel frames were stitched together in post‐image processing of 25 µm Z‐stacks with optical sections at 1.5 µm intervals to detect AF 488, AF 555, and/or AF 647. Counts of tracer‐ or antibody‐labeled neuron groups within the SG were obtained using Imaris 9.7 software (Bitplane, Oxford Instruments, UK). Images were pre‐processed using a set threshold cutoff filter to reduce background on all channels, and somas with an estimated 20 µm diameter were identified and marked via the automated Spots wizard, then reviewed visually for accuracy.

#### 2.7.2 BAT dual‐labeling analysis

To quantify the number of bilateral‐projecting BAT neurons, colocalization of ipsilateral and contralateral tracer within a single cell was determined using the Imaris classification function which automates the detection of cells (marked with Imaris Spots wizard) containing fluorescence from AF 488 or AF 555. Neurons that were dual‐labeled with both fluorophores within SG neurons were then verified by visual examination.

#### 2.7.3 Cell dispersion analysis

To characterize the distribution of cells in the SG, nearest neighbor distances (µm) were measured from the center of each labeled soma to the center of the nearest soma of the same neuron group (e.g., BAT or cardiac; Imaris). The average nearest‐neighbor distance was calculated separately for BAT‐ and cardiac‐projecting SG subpopulations.

#### 2.7.4 Cell volume and morphological analysis

To assess soma volume (µm^3^) and measure primary dendrites and appositions from VAChT inputs, high magnification confocal images were taken of 7–13 cardiac‐ and 7–13 BAT‐projecting cells per SG using 1024 × 1024 pixel images acquired with a Plan‐Apochromat 40×/1.4 Oil DIC (UV) VIS‐IR M27 objective. Neurons were randomly selected by referencing a low magnification image divided into six even sections, and selecting 1–3 cells to image at high resolution from each; more cells were chosen from sections with a higher abundance of neurons. Optical sectioning produced z‐stacks at 0.24 µm intervals that captured the extent of CTb tracer fluorescence in dendritic processes. Analyses were limited to complete CTb‐filled somata that were entirely captured within the XY and Z range of the image stack. Each cell was isolated in a region of interest (ROI) and a consistent smoothing level was applied. The Imaris Surfaces tool calculated the volume of each soma (µm^3^).

The number of primary dendrites per neuron was assessed using Imaris. Primary dendrites were defined as processes branching directly off the soma and extended for a greater distance than the cell body diameter (Ruit & Snider, 1991), excluding the axon. The axon was identified as the least branched and longest process.

Cholinergic inputs on BAT‐ or cardiac‐projecting SG somata were based on the number of vesicular acetylcholine transporter (VAChT) puncta within 0.5 µm of the cell body surface. Criteria for VAChT puncta were: XY diameter >0.4 µm and Z diameter >0.3 µm that spanned more than one optical slice. Intensity threshold cutoffs were set manually based on fluorescence levels in each image. The Imaris Spots tool was used to represent VAChT puncta and an Imaris Xtension MatLab script detected distances between VAChT Spots and soma Surfaces, similar to a protocol described by Slaker et al. (2018). Spots <0.5 µm away from the rendered soma surface were counted as considered apposed to the soma surface.

### 2.8 Statistical analyses

Results are shown as mean ± SEM. Two‐way ANOVAs were used to compare: Cell counts measured by direct tracer visualization versus antibody detection over a time course; cell counts of BAT‐projecting SG neurons over time; cell counts for tracers in left versus right SG; nearest neighbor distance and the number of primary dendrites per cell for BAT and cardiac cells in left versus right SG, with Holm‐Sidak post hoc tests where a significant difference was detected. When no left/right differences were found, SG neurons were grouped according to their target‐specific groups for further analyses. A *t*‐test was performed between BAT‐ and cardiac‐projecting neuron groups to compare cell counts and nearest neighbor distance, or when the data failed a Shapiro–Wilk normality test (*p* < 0.05), a Mann–Whitney rank‐sum test was used in lieu of a *t*‐test (nearest neighbor distance, soma volume, and VAChT normalized for volume). A Pearson product moment correlation was used to examine soma volume and number of primary dendrites, as well as number VAChT puncta per cell for BAT‐ and cardiac‐projecting neurons. A one‐way ANOVA was run on BAT fluorescence intensity at three different time points. Statistical tests were run using SigmaPlot 12.0 (Systat Software). Significance was set at 0.05. In figures containing graphical data, * = *p* < 0.05 and ** =*p* < 0.001.

## 3 RESULTS

### 3.1 Cell detection with CTb tracers is reduced over time

**FIGURE 2:**
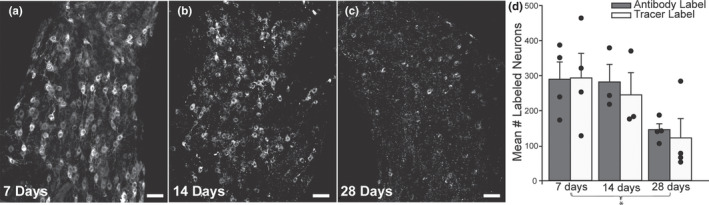
Confocal images of CTb‐labeled BAT‐projecting SG neurons showed reduced detection of cells at 14 and 28 days post‐injection compared to 7 days. (a): BAT‐projecting SG neurons 7 days after tracer injection showed well‐defined cells against a dark background. The cytoplasm contained tracer, while the nucleus was unlabeled. (b): 14 days after injection, fluorescence tended to aggregate in high‐intensity pockets in the cytoplasm of some neurons, and the background contained more punctate non‐specific labeling. (c): 28 days after tracer injection, fluorescence was increasingly punctate and cells were less discernible from the background. (d): Quantification of cell counts showed that both antibody detection (gray bars) and direct visualization of tracer conjugates (white bars) yielded similar cell counts in the SG at each time point. Each dot represents the cell count from 1 SG and each bar represents mean ± SEM (*n* = 4 SG, 2 mice, right and left SG, per post‐injection time). There was a significant decrease in cell counts at 28 days post‐injection compared to 7 days (two‐way ANOVA with post hoc tests, *p* = 0.021). Scale bars =50 µm.

To investigate whether we can use CTb‐conjugated tracers for long‐term tract tracing studies, we examined CTb‐labeled stellate ganglia (SG) at three different time points (7, 14, and 28 days) after injection into brown adipose tissue (BAT) (Figure 1b). One week (7 days) after tracer injections, labeled cells were clearly distinguishable from the background with fluorescence seen throughout the cytoplasm excluding the nucleus (Figure 2a). In contrast, at 14 and 28 days following tracer injections we found the fluorescence tended to aggregate in high‐intensity pockets in the cytoplasm rather than filling the cytoplasm (Figure 2b,c) making the boundaries of cell bodies less defined, leading to reduced cell number detection at later time points (Figure 2d).

To determine if we could better amplify tracer‐filled cells, we used antibody detection of CTb (Hegarty et al., 2014) and compared cell counts using both detection methods in the same ganglion (with different fluorophores on the CTb tracer and the secondary antibody to CTb). We compared cell counts from direct tracer visualization via the conjugated fluorophore to the antibody detection of CTb in BAT‐injected mice (2 male mice per post‐injection time, 29 weeks, 29–39 g). At each post‐injection time, the antibody‐label and tracer‐conjugated fluorophores were colocalized in the same cells, and yielded similar cell counts (Figure 2d). Reflecting our qualitative observations, cell counts (mean ± SEM) of tracer‐ and antibody‐labeled neurons were lower in SG at later time points (Figure 2d; 7 days: Antibody: 290 ± 49 cells tracer: 294 ± 70 cells; 14 days: Antibody: 282 ± 50 cells, tracer: 245 ± 110 cells; 28 days: Antibody: 146 ± 17, tracer: 122 ± 55 cells). Cell counts were significantly lower at 28 days post‐injection compared to 7 days.

To uncover whether the reduction in cell detection was due to a loss in fluorescence intensity, we sampled the mean intensity (pixels) of 10–12 tracer‐labeled BAT neurons from each SG at 7, 14, and 28 days post‐injection. The mean ± SEM intensity decreased with longer post‐injection periods (7 days: 6684 ± 1843 pixels; 14 days: 4268 ± 281 pixels; 28 days: 2999 ± 858 pixels), but this reduction was not statistically significant (one‐way ANOVA). These findings suggest that some loss of fluorescence intensity combined with the intracellular aggregations of the fluorescent conjugate led to reduce high fidelity detection of labeled neurons over time.

### 3.2 BAT SG innervation is predominantly ipsilateral

**FIGURE 3:**
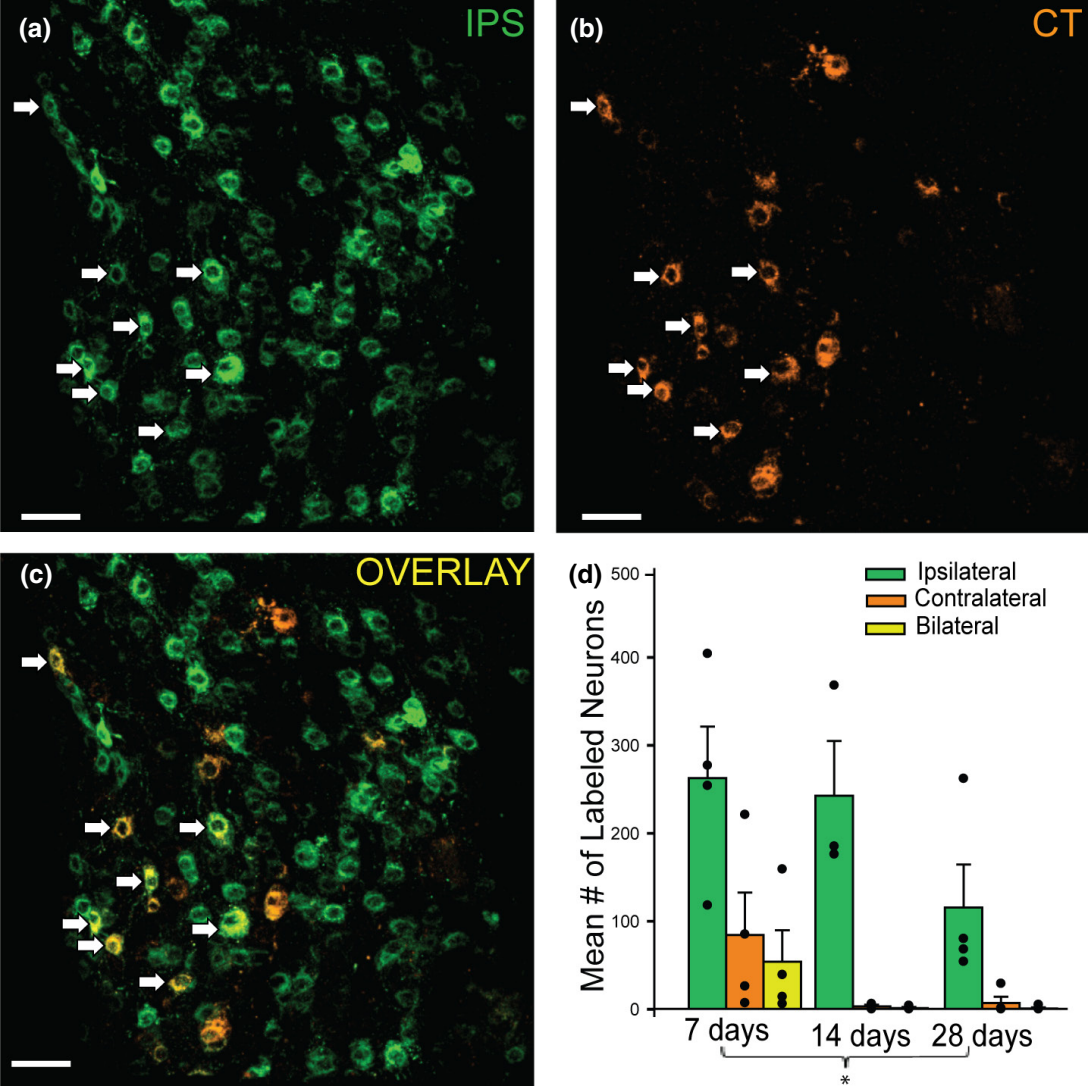
BAT‐projecting SG neurons primarily innervate the ipsilateral side, but some SG neurons project bilaterally to BAT. (a): A confocal image of BAT‐projecting SG neurons (green) labeled following an ipsilateral (IPS) injection. (b): A confocal image of neurons (orange) labeled following a contralateral (CT) BAT injection in the same animal. There were fewer contralateral BAT‐projecting neurons in the SG than ipsilateral BAT‐projecting neurons. (c): A confocal image overlay of ipsilateral‐ and contralateral‐BAT injected channels showing some SG neurons that were dual‐labeled (yellow) indicating they project bilaterally to intrascapular BAT. White arrows in all panels show bilateral‐projecting BAT neurons in the SG. (d): Cell count results for ipsilateral‐, contralateral‐, and bilateral‐projecting BAT neurons at 7, 14, and 28 days after tracer injections, each dot represents a cell count from 1 SG and each bar represents mean ± SEM (*n* = 4 SG, 2 mice, right and left SG, per post‐injection time). There were significantly more BAT‐projecting SG ipsilateral neurons at each time point (two‐way ANOVA with post hoc tests, *p* < 0.001), and there was a significant decrease in overall tracer‐labeled BAT‐projecting SG neurons at 28 days compared to 7 days after injection (*p* = 0.019). Note, the same mice were used for the experiment described in Figure 2D, which reports the total tracer‐labeled cells regardless of right or left tracer injection. Scale bars =50 µm.

To assess the circuitry of the BAT‐projecting SG subpopulations, left and right intrascapular BAT deposits were injected with different CTb tracer conjugates (AF 488 and AF 555) in a counterbalanced fashion (Figure 1b). Ipsilateral‐ (IPS), contralateral‐ (CT), and bilateral‐ (BI) projecting cell counts were assessed in SG harvested 7, 14, and 28 days after tracer injections (Figure 3). With our tracing method, ipsilateral‐projecting BAT neurons were most abundant (Figure 3a), while some SG neurons projected to the contralateral BAT (Figure 3b). A small number of BAT neurons projected bilaterally to both left and right BAT, showing co‐localization of both tracer conjugates (Figure 3c). These results (mean ± SEM) were quantified for the 7 day time point (Figure 3d) (IPS: 263 ± 59 cells, CT: 85 ± 48 cells, BI: 55 ± 36 cells). Consistent with the time course results, fewer SG neurons projecting ipsilaterally, contralaterally or bilaterally to BAT were detected at 28 days versus 7 days post‐injection (Figure 3d).

### 3.3 BAT‐ and cardiac‐projecting SG neurons are separate subpopulations

**FIGURE 4:**
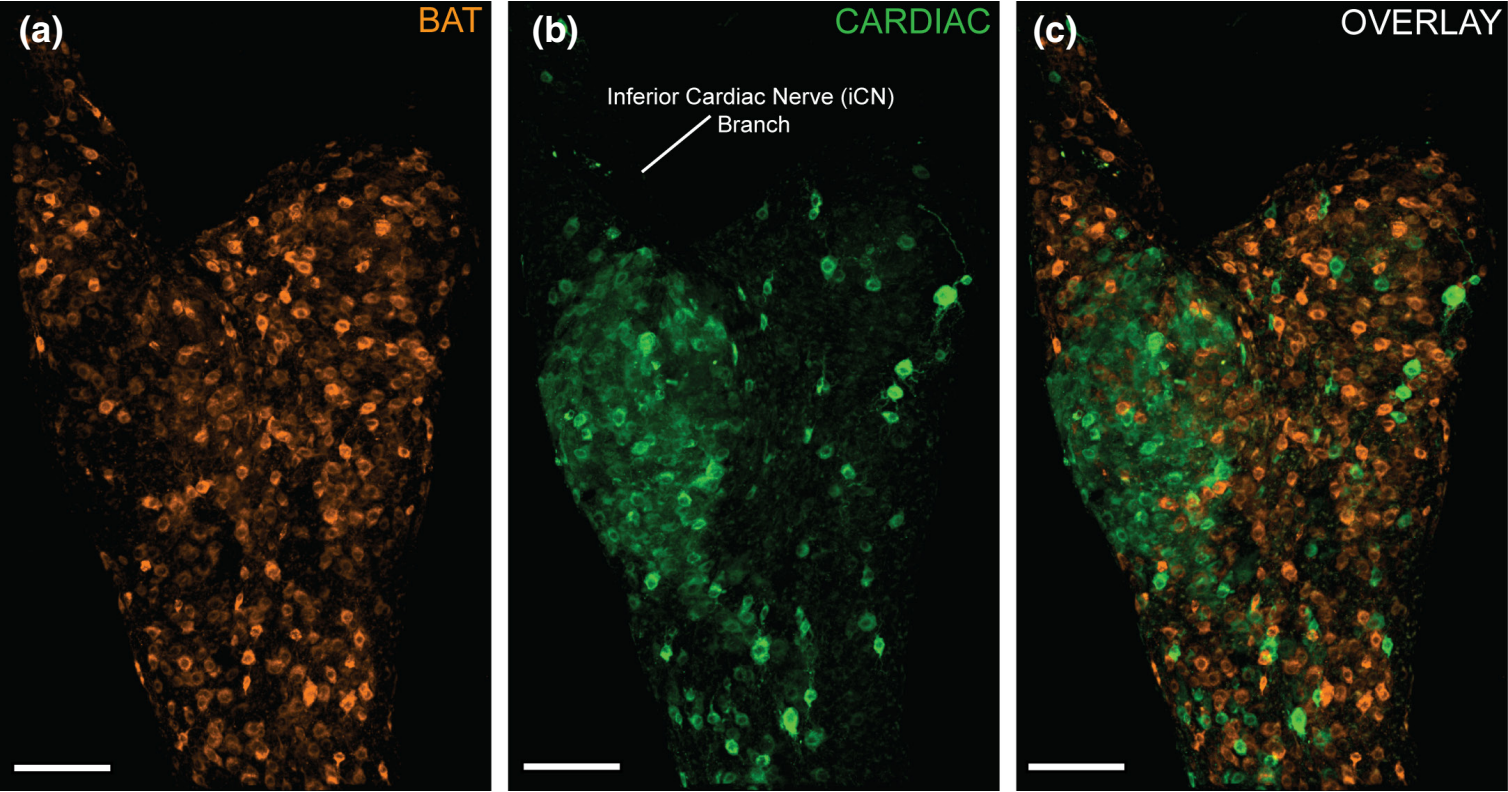
Dual tracing demonstrates that BAT‐ and cardiac‐projecting SG neurons are distinct subpopulations. Confocal images of a left SG are shown from the supine perspective. (a): BAT‐projecting SG neurons (orange cells, CTb AF 555) are widely distributed throughout the SG. (b): Cardiac‐projecting neurons (green cells, CTb AF 488) are distributed particularly at the base of the inferior cardiac nerve (iCN) branch. (c): Both BAT‐ (orange) and cardiac‐ (green) projecting neurons are shown in the overlay confocal image, and there was no colocalization of tracers, indicating these are distinct groups of neurons. Scale bars =100 µm.

To compare BAT and cardiac SG subpopulations, we injected different tracer conjugates (AF 488 and AF 555) into BAT and the pericardium, counterbalanced tracers between sites (Figure 1c two females and two males, 27–32 weeks, 24–32 g mice) and observed retrogradely labeled SG neurons at 7 days post‐injection (the time point when we found the most robust retrograde tracer labeling following BAT injections, Figure 2) (Figure 4). While BAT‐projecting cells were widely dispersed throughout both ganglia (Figure 4a), cardiac‐projecting cells were primarily clustered at the base of the inferior cardiac nerve (iCN) branch, which extends medially in the direction of the heart (Figure 4b). In dual‐labeled animals, we did not detect any SG neurons that project to both BAT‐ and the pericardium (Figure 4c).

### 3.4 BAT‐projecting SG neurons are more populous than cardiac‐projecting SG neurons

**FIGURE 5:**
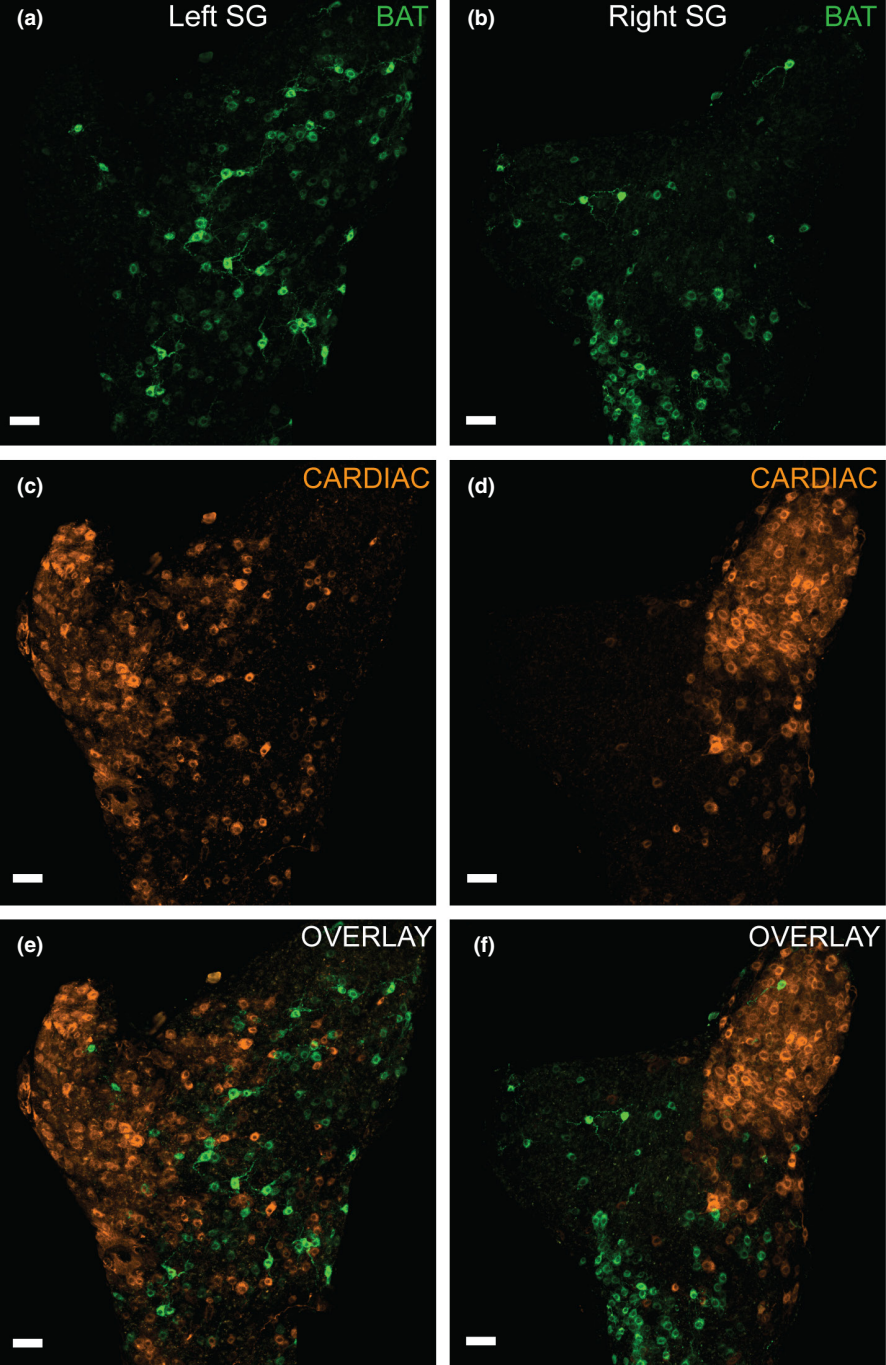
Confocal images of BAT‐ (green cells) and cardiac‐ (orange cells) projecting neurons for left and right SG are shown. Each ganglia is oriented as if the mouse was prone. (a and b): BAT‐projecting SG neurons (green cells) retrogradely labeled with CTb‐ AF 488 in left (a) and right (b) SG. (c and d): Cardiac‐projecting neurons (orange cells) labeled with CTb‐ AF 555 in left (c) and right (d) SG, and are concentrated at the base of the inferior cardiac nerve in both ganglia. (e and f): The overlay shows that BAT‐ and cardiac‐projecting subpopulations are distinct and do not contain dual‐labeled cells in left and right SG. Scale bars =70 µm.

**FIGURE 6:**
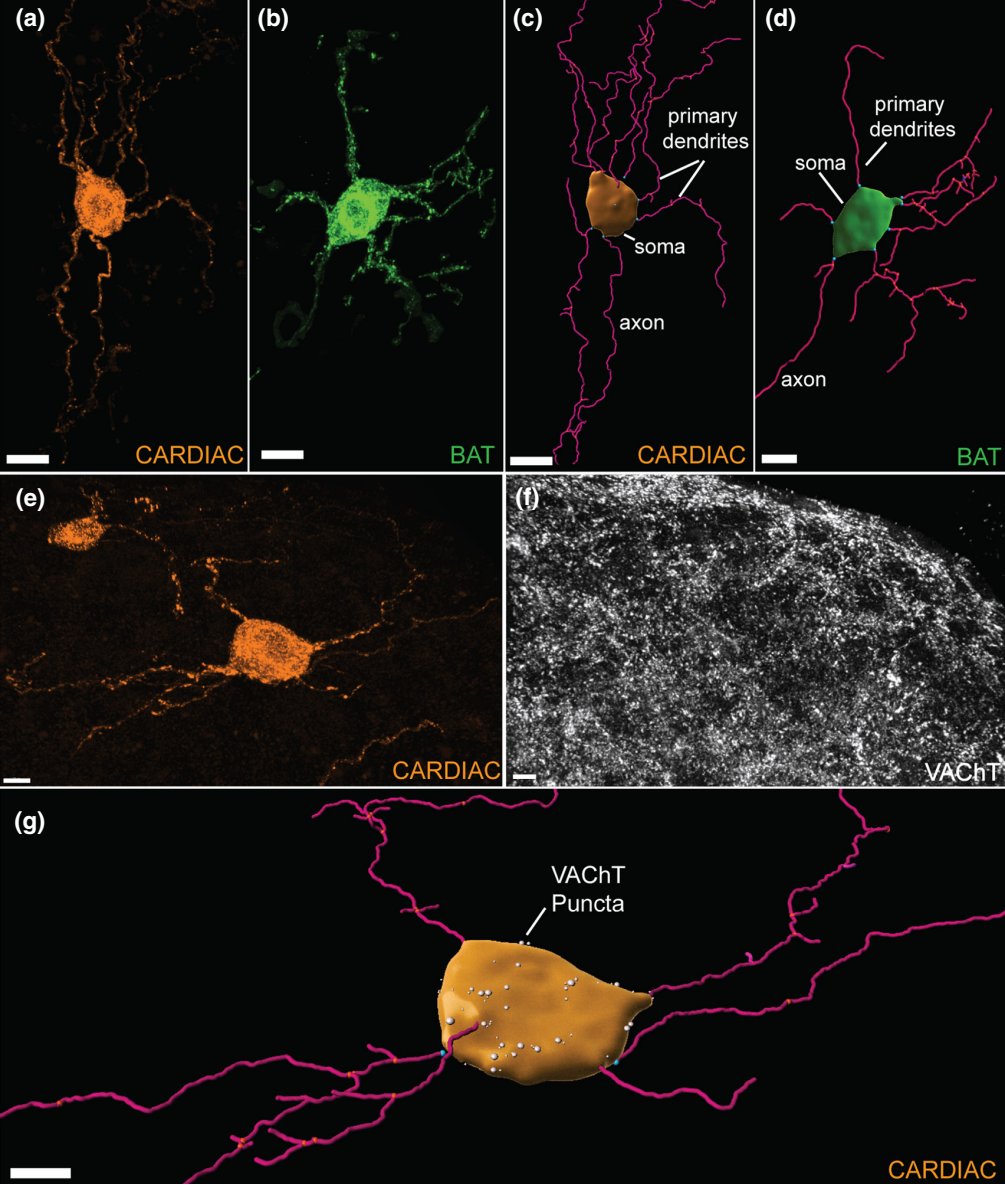
Representative confocal images and 3D reconstructions of stellate ganglia (SG) sympathetic neurons. (a): Confocal image of a cardiac‐projecting SG neuron (scale bar =15 µm). (b): Confocal image of a BAT‐projecting SG neuron (scale bar =20 µm). A region of interest and selective masking was used to isolate single neurons in a confocal scan for analysis. (c): A 3D reconstruction of the cardiac‐projecting neuron (a); (d): 3D reconstruction of the BAT‐projecting neuron (b). (c and d): Imaris Surfaces‐ and Filaments‐generated objects modeled soma and dendrites. Each primary dendrite branch point is labeled blue. The soma volume (µm^3^) and number of primary dendrites of each cell was assessed. (e): A confocal image of a cardiac‐projecting SG neuron. (f): A confocal image showing vesicular acetylcholine transporter (VAChT) puncta in SG. (g): Imaris reconstructions of a cardiac‐projecting neuron and VAChT puncta (white spots) that were apposed to the soma surface. Scale bars for E, F, and G = 10 µm.

**FIGURE 7:**
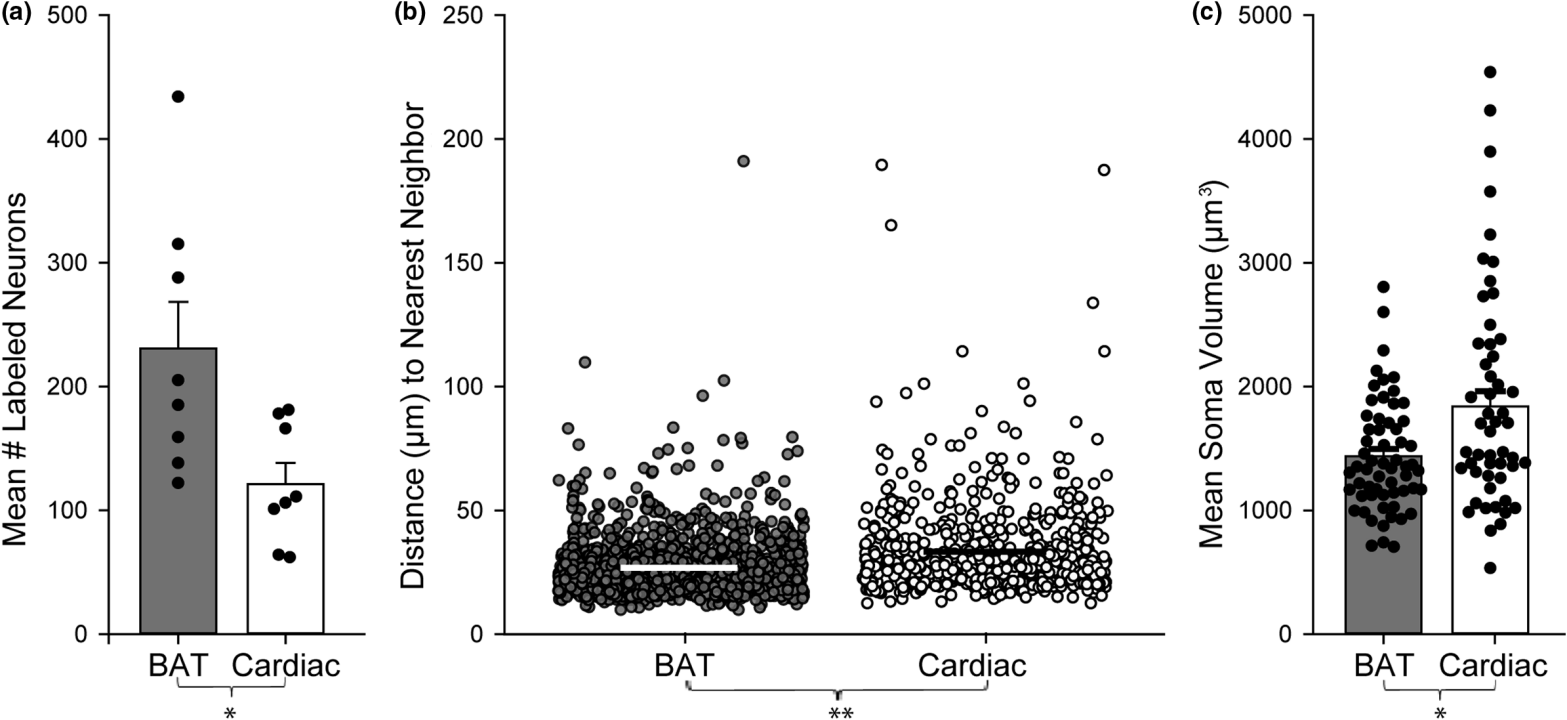
Several differences were seen between BAT‐ and cardiac‐projecting SG neuron subpopulations. (a): There were significantly more BAT‐projecting neurons (grey bar) than cardiac‐projecting neurons (white bar) within the SG in our dual‐labeled animals (*n* = 8 SG, 2 males and 2 females, right and left SG, *t*‐test, *p* = 0.019). Each dot represents a subpopulation cell count from one SG and the bar represents the mean ± SEM. (b): For each SG neuron, we measured the distance to the nearest neighbor within that subpopulation (e.g., BAT‐to‐BAT or cardiac‐to‐cardiac). The distance is plotted for each neuron, the x‐axis was expanded to allow visualization of all counted cells. Overall, BAT‐projecting neurons (grey circles) had a mean nearest neighbor distance (µm) (mean ± SEM is white horizontal bar) that was smaller than cardiac‐projecting cells (white circles, mean ± SEM is black horizontal bar) (*n* = 1338 BAT‐ and 633 cardiac‐projecting SG cells, 3 mice, 6 right and left SG, 1 male and 2 females, Mann–Whitney rank‐sum test, *p* < 0.001). (c): BAT‐projecting SG somata (grey bar) volumes (µm^3^) are less than cardiac‐projecting SG somata volumes (white bar) (*n* = 61 BAT and 54 cardiac‐projecting SG cells, 3 mice, 6 right and left SG, 1 male and 2 females, Mann–Whitney rank‐sum test, *p* = 0.022). Each dot represents the soma volume of one neuron and the bar represents the mean ± SEM, showing a wide range of soma sizes for cardiac‐projecting neurons, compared to a smaller array for BAT‐projecting neurons. Volume was calculated from Imaris neuron reconstructions (see Figure 6).

Right and left SG displayed similar dispersion patterns (Figure 5) for both BAT and cardiac targets, and the cell counts of BAT and cardiac subpopulations did not differ significantly between left and right SG (Figure 5, two‐way ANOVA). Since we did not find left/right differences, left and right SG cell populations were grouped for further analyses. For tracer‐labeled SG subpopulation cell count and dispersion, confocal images of the entire ganglion were assessed (Figures 4, 7), while structural analyses were conducted using Imaris reconstructions of isolated BAT‐ and cardiac‐projecting neurons imaged at higher magnification (Figures 6, 7). Overall, we found more BAT‐projecting neurons than cardiac‐projecting neurons within the SG (Figure 7a, Table 1) using our tracing paradigm, however, the distribution and morphology of these two populations had other distinct features (see below).

**TABLE 1:** Morphological features of BAT‐ and cardiac‐projecting stellate ganglion neuron subpopulations

	BAT	Cardiac
Cell count	231 ± 38 cells*	121 ± 17 cells*
Nearest neighbor (µm)	27.2 ± 0.3 µm**	33.7 ± 0.8 µm**
Soma volume (µm^3^)	1437.1 ± 57.3 µm^3^ *	1839.1 ± 123.6 µm^3^ *
Primary dendrites/cell	3.0 ± 0.1	3.0 ± 0.2^a^
VAChT puncta/cell	26.2 ± 2.7 puncta^a^	30.7 ± 4.4 puncta^a^
VAChT puncta/1000 µm^3^ (normalized for cell volume)	18.3 ± 1.7 puncta	17.1 ± 2.2 puncta

Note

All values listed are mean ± standard error (SEM).

^a^ Correlated with soma volume.

* *p* < 0.05.

** *p* < 0.001.

### 3.5 BAT‐ and cardiac‐projecting SG subpopulations have different dispersion patterns

We observed an asymmetrical clustering of cardiac‐projecting SG neurons at the base of the iCN branch, in contrast with a symmetrical dispersion pattern of BAT‐projecting neurons throughout the SG using our tracing method (Figure 4). To quantify dispersion, nearest neighbor measurements (µm) were collected for each neuron within that subpopulation, and averaged for BAT‐ and cardiac‐projecting SG neurons. BAT‐projecting SG neurons had a mean distance to their nearest neighboring cell that was smaller than cardiac‐projecting SG neurons (Figure 7b, Table 1). We found no difference in nearest neighbor distance between right and left SG for each population, indicating that left and right SG dispersion of both subpopulations within each ganglion was similar (two‐way ANOVA). This is consistent with our observations of the dispersed (BAT) and clustered (cardiac) dispersion patterns of these sympathetic neuron SG subpopulations (Figure 4).

### 3.6 Cardiac‐projecting SG somas are larger than BAT‐projecting SG somas

To compare morphology between BAT‐projecting and cardiac‐projecting SG neurons, we did 3D reconstructions (Figure 6a–d) of 54 cardiac‐ and 61 BAT‐projecting somata from dually‐labeled mice (1 male and 2 females, 27–32 weeks, 24–30 g). We measured soma volume (µm^3^) and number of primary dendrites of each neuron (Figure 6a–d). There was no statistical difference between left and right SG soma volumes within both neuron subgroups (two‐way ANOVA), so they were combined for further analysis. Cardiac‐projecting somata had more volume than BAT‐projecting somata (Figure 7c, Table 1), although it was notable that the BAT cell bodies were more uniform in size than cardiac‐projecting SG neurons (Figure 7c). No sex differences were found for BAT‐ or cardiac‐projecting SG soma size, primary dendrite number per cell and cholinergic appositions on the soma (two‐way ANOVA).

### 3.7 Cardiac‐projecting SG soma volume is positively correlated with number of primary dendrites per cell

**FIGURE 8:**
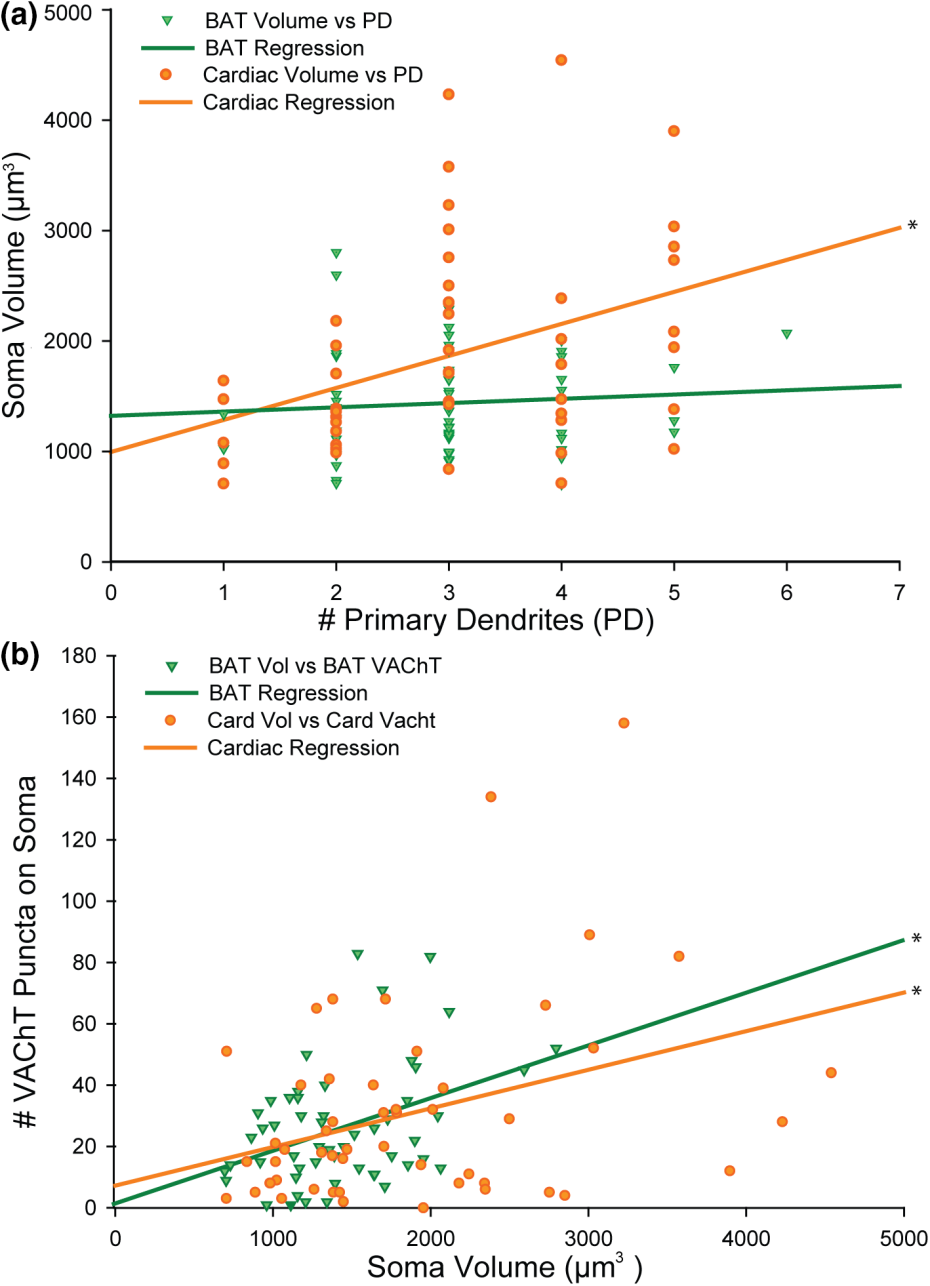
Soma size is related to the number of primary dendrites for cardiac‐ but not BAT‐projecting neurons (a) while VAChT puncta are related to cell volume for both cell types (b) (*n* = 3 mice, 6 SG, 2 males, and 1 female, Pearson product moment correlations). (a): Cardiac‐projecting SG neuron soma volume (µm^3^) is positively correlated to the number of primary dendrites per cell (orange circles, 54 cells analyzed, orange regression line, *r* = 0.357, *p* = 0.008); there was not a significant correlation between these factors for BAT‐projecting SG neurons (green triangles, 61 cells analyzed, green regression line, *r* = 0.086). (b): In both BAT‐ and cardiac‐projecting subpopulations, the number of vesicular acetylcholine transporter (VAChT) puncta apposed to the soma surface per cell is positively correlated to the soma volume (µm^3^) (BAT: Green triangles, 53 cells analyzed, green regression line, *r* = 0.404, *p* = 0.003; cardiac: orange circles, 53 cells analyzed, orange regression line, *r* = 0.354, *p* = 0.009).

To further elucidate the morphology of each SG subpopulation, we counted the number of primary dendrites for individual neurons using Imaris (Figure 6a–d). No difference was found in the number of primary dendrites per cell between BAT‐ and cardiac‐projecting SG neurons (Table 1, two‐way ANOVA). However, the number of primary dendrites per cell was positively correlated to soma volume (µm^3^) in the cardiac‐projecting SG subpopulation, with larger cells having more primary dendrites (Figure 8a). Interestingly, these factors were not correlated in BAT‐projecting SG neurons (Figure 8a).

### 3.8 VAChT apposition number increases with soma volume

To examine cholinergic inputs to SG neurons, we combined immunocytochemical detection of acetylcholine transporter (VAChT) with detection of retrogradely labeled neurons in SG. VAChT puncta apposed to either BAT‐ or cardiac‐projecting SG neuron somata were imaged via high‐resolution confocal microscopy and then VAChT puncta were 3D‐rendered with Imaris (Figure 6e–g). The number of puncta apposed to the soma surface was similar for both BAT‐ and cardiac‐projecting SG subpopulations (Table 1, two‐way ANOVA) and both had positive correlations with soma volume (µm^3^) (Figure 8b). Before or after normalizing the data for each neuron's soma volume (number of VAChT puncta/1000 µm^3^), we found no difference in VAChT input between BAT and cardiac subpopulations (Table 1, Mann–Whitney rank‐sum test).

## 4 DISCUSSION

We have shown that BAT‐ and cardiac‐projecting neuronal populations in the mouse stellate ganglia (SG) are distinct, non‐overlapping neurons that are morphologically different. BAT‐projecting neurons are greater in number and found more diffusely in both left and right stellates with our labeling methods. While BAT‐projecting neurons are found primarily ipsilateral to the fat pad that they innervate, some project to the contralateral side and a small subset project bilaterally. In contrast, cardiac‐projecting neurons were larger, and were found in a more restricted region of the stellate.

We have demonstrated the ability to identify distinct target‐specific populations of SG neurons, which reflects the traditional knowledge of segmented sympathetic outflow to target tissues (Wiedmann et al., 2017). In a previous study, target‐specific neuron groups of the left superior cervical sympathetic ganglia were identified via retrograde tracing from three separate target tissues in a study on the rat. That study also found the neuron populations to be morphologically different (Andrews et al., 1996), however, only one target was injected per animal, while our method used two CTb fluorophore conjugates to label multiple neuron subpopulations within the same animal, thus cementing the notion of sympathetic innervation that is target‐specific.

A marked decrease in cell detection using CTb fluorophore conjugate tracers was evident over time after injection into BAT. CTb is known to retrogradely transport in 2–7 days (Saleeba et al., 2019); however, we have not found previous studies detailing the stability of the tracer in rodents at later time points. At 1 week, smooth cytoplasmic and dendritic labeling is distinct from the background, but at later time points, aggregates of fluorescence make cell features less discernable compared to the background. Using antibody labeling for CTb, which was historically a method to visualize the tracer (Aicher et al., 2013; Hegarty et al., 2018; Saleeba et al., 2019), we ruled out the possibility that the fluorophore was detaching over time and concluded that the CTb tracer itself may be aggregating in subcellular structures. Despite this limitation, our retrograde labeling approach remains the most suitable for the analysis of multiple groups of neurons based on their target tissue, without the use of viruses and genetic manipulations that may alter the structure and function of transfected cells (Kumar, 2019).

Injections in both the pericardium and BAT produced consistent, bright, and photostable SG neuronal labeling across multiple animals. CTb‐conjugated fluorophores are known to be readily taken up by and retrogradely transported from axon terminals, even when injected into a confined site (Conte et al., 2009). Even so, it should be noted that retrograde tracing may not label the entirety of a neuronal subpopulation, and our cell counts for BAT‐ and cardiac‐projecting SG neurons are slightly lower compared to previous reports in mice (Francois et al., 2019; Rajendran et al., 2019). This may partly be explained by which regions were included in previous analyses. For example, Francois et al. counted all BAT‐projecting cells in the SG and T1 ganglia combined, while we only counted SG proper. It is always important to note that our interpretations are limited to the cells that we are able to identify and label, recognizing that we will not be identifying the entire population of projection neurons. Regardless of overall numbers, our approach offers a robust method to allow comparisons of cell numbers and dispersion of two tissue‐specific neuron groups in the SG.

We tailored our injection methods for each site to reduce the risk of tracer leaking to nearby organs, while accurately representing sympathetic innervation. BAT is more porous than the pericardial sac, so we injected a smaller volume in multiple locations on the right and left fat pads. In contrast, the pericardial sac was effective in keeping the injectate localized to the heart, so we injected more volume than in BAT through 2–3 holes to allow diffusion over a large region of the left ventricle. Both strategies produced consistent, bilateral SG labeling in each mouse. Despite lower total volume of tracer injectate in BAT, we consistently found more BAT‐projecting neurons in SG than cardiac‐projecting neurons.

We found BAT‐projecting neurons primarily in the SG ipsilateral to the injection site, which aligns with a recent study on sympathetic BAT innervation using a pseudorabies virus unilateral BAT infection in mice (Francois et al., 2019). It has traditionally been thought that each BAT pad is innervated exclusively by ipsilateral sympathetic efferents (Bartness et al., 2010; Foster et al., 1982; Hamilton et al., 1989). This has been determined by assessing norepinephrine levels after unilateral or bilateral sympathetic denervation to intrascapular BAT in rats (Foster et al., 1982) and hamsters (Hamilton et al., 1989). Our bilateral injection approach employed the use of two CTb tracer fluorophore conjugates injected in right and left BAT depots. It is worth noting that exclusively male mice were used to assess CTb tracer stability and BAT‐projecting neuron circuitry over a time course. For the purpose of this experiment, sex ratio should have minimal impact on results. Sex differences in SG gene expression have been found in previous studies (Bayles et al., 2018, 2019), and we use both male and female mice for morphology determinations of both BAT‐ and cardiac‐projecting SG neurons. Using our dual tracing method, we elucidated the existence of two smaller, but distinct, subsets of SG neurons that project contralaterally and bilaterally to BAT. We don't believe this to be a consequence of diffusion to the contralateral BAT, as we consistently observed this across multiple animals with both tracer conjugates.

Cardiac‐projecting SG neurons were found bilaterally after pericardial injections with no significant left/right differences. Prior studies in the rat (Pardini et al., 1989; Yasunaga & Nosaka, 1979) and mouse (Rajendran et al., 2019) have also found these neurons to project bilaterally to the heart. Previous publications have also reported differential and specific control of the heart from left and right SG (Ardell et al., 1988; Lujan et al., 1985; Salo et al., 2006), however, these studies were done in other species, including functional studies in humans (Zandstra et al., 2021), and may not be apparent in the mouse. Although we did not find structural left/right differences, it is possible functional differences are present in the mouse; however, prior studies have suggested that sympathetic neuron morphology tends to correspond with function (Jobling & Gibbins, 1999).

Cardiac‐projecting neurons had larger cell bodies than BAT‐projecting neurons. This finding is consistent with prior studies indicating that subtypes of neurons may be distinct and that morphology may be partly regulated by target tissues (Andrews et al., 1996). Our findings of larger cell bodies for cardiac‐projecting neurons are in contrast to one previous report that found no differences between cardiac and non‐cardiac neurons (Mo et al., 1994), however, that study identified subtypes only from which branch they followed out of the ganglion, sampled smaller numbers of cells, was conducted using only area measurements in two dimensions, and was done in rats rather than mice. We found that while cardiac‐projecting SG neurons were on average larger, the range of sizes and numbers of dendrites shared some overlap with BAT‐projecting cells.

Post‐ganglionic neurons of the sympathetic chain, including both types of SG cells, receive cholinergic inputs (Landis et al., 1987), which we examined by antibody labeling VAChT appositions on the cell body surface. While cardiac‐projecting neurons had slightly higher numbers of inputs, the BAT‐ and cardiac‐projecting SG cell input did not vary dramatically. Both cell types’ inputs were positively correlated with soma size, suggesting that cholinergic inputs have similar density around the somatic membrane. The topography of this input is likely regulated by developmental factors (Scott‐Solomon & Kuruvilla, 2018), and target‐derived neurotrophic signaling (Sharma et al., 2010). Our findings are consistent with a previous study that showed larger sympathetic cell bodies with greater dendritic complexity received more pre‐ganglionic axonal inputs (Purves & Lichtman, 1985), which may explain the correlation of VAChT inputs to soma volume we observed in BAT‐ and cardiac‐projecting SG cells.

Our data show the same mean number of primary dendrites per neuron for BAT‐ and cardiac‐projecting SG neurons. Multiple previous publications in the rat (Andrews et al., 1996) and mouse (Ruit et al., 1990) studying superior cervical sympathetic ganglion cells also found target‐specific neuron groups to have a similar number of primary dendrites per cell. Similarly, these studies reported that neuron structural metrics varied significantly between target‐specific neuron groups including dendrite length, area, and soma size, but interestingly not the number of primary dendrites.

Although we found both populations of SG neurons to have similar numbers of primary dendrites, we found differences when correlating the number of primary dendrites to soma size. We found a positive correlation between soma size and the number of primary dendrites in cardiac‐projecting SG neurons, but not in BAT‐projecting SG neurons which may be related to target tissue‐derived signaling. Sympathetic neuronal plasticity has been shown to be modulated by neurotropic signaling from the innervated tissue (Andrews et al., 1996; Ernsberger & Rohrer, 2018; Sharma et al., 2010). A commonly studied neurotrophin, nerve growth factor (NGF), has proven to be essential for the construction and maintenance of pre‐ and post‐ganglionic synapses (Sharma et al., 2010). Neural plasticity in sympathetic ganglia has also been observed after injury in studies performed on larger mammalian species (Ajijola et al., 2015; Nguyen et al., 2012). One study examined this following a T5 spinal cord transection in rats, which is known to cause abnormal sympathetic regulation of heart rate and cardiac function. Nerve growth factor (NGF) in the left ventricle and SG was found to be elevated, and post‐ganglionic SG neuron dendritic branching increased after spinal cord transection (Lujan et al., 1985, 2010). In another study in the mouse, daily NGF administration triggered soma growth in SG neurons, and increased dendritic branching in superior cervical ganglion neurons. This study also did not find primary dendrite number to significantly increase with NGF treatment (Ruit et al., 1990). This may help explain the morphological differences we have found between both SG neuron types, though further exploration is needed.

Our method of identifying SG sympathetic neurons via retrograde fluorophore conjugates has allowed for the examination of two non‐overlapping subpopulations of cells. We have found cardiac‐projecting SG cells to be larger and localized, while BAT‐projecting SG neurons are smaller and evenly dispersed in the ganglia. Both subpopulations have similar numbers of primary dendrites per cell, and similar densities of cholinergic inputs on their cell bodies. Through this comparative approach of retrograde labeling from target tissues, we have developed a deeper understanding of the circuitry and morphology of BAT‐ and cardiac‐projecting SG neuron subpopulations.

## AUTHOR CONTRIBUTIONS

Madeleine S. Barrett carried out experiments; Sue A. Aicher, Deborah M. Hegarty, and Madeleine S. Barrett designed experiments; Madeleine S. Barrett conducted data analysis; Madeleine S. Barrett, Deborah M. Hegarty, Beth A. Habecker, and Sue A. Aicher wrote and edited the manuscript.

## ACKNOWLEGMENTS

The authors are grateful to outstanding staff at the OHSU Advanced Light Microscopy Core for their guidance, particularly Brian Jenkins and Stefanie Kaech Petrie. We also thank Lori M. Zeltser, Ph.D. at Columbia University for graciously sharing a surgical protocol with us.

## CONFLICT OF INTEREST

The authors have no competing interest to declare.

## ETHICS STATEMENT

All experiments were approved by the Oregon Health & Science University (OHSU) Institutional Animal Care and Use Committee.
